# Long-term survival of a patient with lung cancer metastasis to the spine following surgical treatment combined with radiation and epithelial growth factor receptor inhibitor therapy: A case report

**DOI:** 10.3892/etm.2014.2055

**Published:** 2014-11-07

**Authors:** SONGFENG XU, XIUCHUN YU, MING XU

**Affiliations:** Department of Orthopedics, General Hospital of Ji’nan Military Region, Ji’nan, Shandong 250031, P.R. China

**Keywords:** lung cancer, spine metastases, surgical treatment, radiation, epidermal growth factor receptor inhibitor

## Abstract

The prognosis of patients with lung cancer metastasis to the spine is poor, and the choice of surgery is questionable based on the aggressiveness of the disease. The present study describes a case of a 56-year-old male with metastatic spinal cord compression. The patient underwent surgery for posterior decompression and internal fixation, in addition to receiving postoperative radiation and epidermal growth factor receptor (EGFR) inhibitor medication. After 24 months, positron emission tomography-computed tomography scan showed a reduction in the left upper lobe mass in the short axis and inactivation of the neoplasm in the left upper lobe and T9 vertebra. Based on these promising results, it is suggested that orthopedic oncologists consider the combination of radiation and EGFR inhibitor therapy with surgery for the treatment of lung cancer metastasis to the spine.

## Introduction

The prognosis of patients with lung cancer metastasis to the spine is poor, and the option of surgery is questionable based on the aggressiveness of the disease. Between 5 and 10% of patients with systemic cancer develop vertebral metastases (1–4). The thoracic spine is the most common region involved in spinal metastases (70%) (5). The five-year survival rate for patients with lung cancer is 10 to 20%, as reported by Sundaresan *et al* (2) Stanley (6) and Freise *et al* (7) indicating a poor prognosis. In a retrospective study of 118 patients with bone metastasis from lung cancer, treatment with an epidermal growth factor receptor (EGFR) inhibitor indicated improved prognosis for patients with adenocarcinoma. The mean survival time was longer in the group treated with the EGFR inhibitor (17.8 months; range, 8.4–30.1 months) than that in the group without the EGFR inhibitor (10.8 months; range, 0.6–54.0 months) among 52 patients with adenocarcinoma metastases (8). It is therefore possible that the survival time for certain patients could be extended following appropriate surgical treatment combined with radiation and EGFR inhibitor therapy.

## Case report

**Figure 1:**
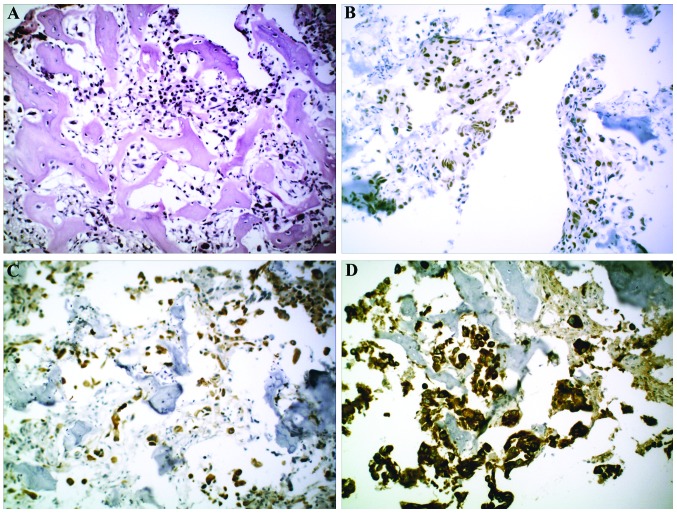
Postoperative immunological pathology findings confirmed the lesion to be non-small cell lung cancer (adenocarcinoma). (A) Hematoxylin and eosin staining. (B–D) Immunological images showing the lesion to be (B) thyroid transcription factor 1-positive, (C) cytokeratin (CK) 88-positive and (D) CK-positive. All images: Magnification, ×200.

The present study describes the case of a 56-year-old male who seeked consultation due to back pain [Visual Analog Scale (VAS) of 7/10], numbness below the umbilicus and weakness of the bilateral lower limbs. The patient had no other signs or symptoms, no significant past medical history and no history of smoking. The patient was immediately referred to the Department of Orthopedic Oncology of the General Hospital of Ji’nan Military Region (Ji’nan, China). The laboratory findings were unremarkable. Upon arrival to the inpatient department, a computed tomography (CT) scan and X-ray of the spine were taken. An abnormal mass was noted at the level of T9 and the left upper lobe of the lung. Further investigation with magnetic resonance imaging (MRI), positron emission tomography (PET)-CT and biopsies of the vertebral and pulmonary lesions confirmed the diagnosis of a single metastatic lesion at the level of T9. The primary site was the lung with staging of T1N0M1. The lesion was confirmed to be non-small cell lung cancer (adenocarcinoma) with immunological pathology (Fig. 1). A mutation to the EGFR gene was identified (deletion of exon 21). The MRI showed pedicle involvement and spinal canal stenosis. The vertebral lesion spanned the entire T9 vertebra. The Tokuhashi score (9) was nine, which indicated survival of >6 months, and palliative surgery was recommended.

**Figure 2:**
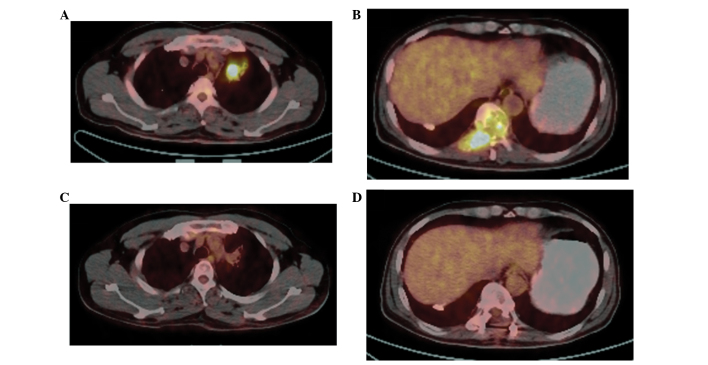
(A and B) PET-CT scan showed an abnormal mass at the level of T9 and the left upper lobe of the lung. (C and D) Twenty-four months after treatment, PET-CT scan showed inactivation of the neoplasm in the left upper lobe and T9 vertebra and (C) a decrease in the left upper lobe mass in the short axis. PET-CT, positron emission tomography-computed tomography.

The surgery consisted of a posterior decompression with partial resection of the T9 tumor followed by the installation of a posterior pedicle screw system between T7 and T11. The patient fully recovered and had no complications due to surgery. His postoperative VAS was reduced to 2/10 and the numbness and weakness disappeared immediately subsequent to the surgery. The patient received follow-ups at 1, 3, 6, 12 and 24 months without recurrence of the numbness or weakness or any other complication. The patient was also treated with three cycles of three-dimensional (3D) conformal radiotherapy (RT) (30 cGy/day for 10 days) localized to the spine and lung subsequent to surgery. The RT was completed without any associated complications. Erlotinib therapy (150 mg daily) was initiated from February 2012, and continues to date, and the patient experienced significant symptom improvement. After 24 months, PET-CT scan showed a decrease in the left upper lobe mass in the short axis and inactivation of the neoplasm in the left upper lobe and T9 vertebra (Fig. 2). This patient continues to be monitored through follow-up appointments. Informed consent was obtained from either the patient or the patient’s family prior to inclusion in the present study.

## Discussion

As well as chronic and increasing pain, spinal metastases cause neurological deficits due to destruction of the vertebral body and subsequent epidural growth expansion (10). The primary aim of surgery in the treatment of spinal metastases is the reduction of pain and the maintenance of neurological function and spinal stability. The decision to proceed with surgery should be determined individually in an interdisciplinary consultation. In a previous retrospective cohort study of 2,321 consecutive patients with acute symptoms of metastatic spinal cord compression (MSCC) admitted to a single center, the patients with MSCC deriving from pulmonary and renal cancer experienced an improved one-year survival compared with other oncologic diagnoses (11). The selection of surgical treatment for MSCC has increased due to positive results from clinical studies, improved surgical techniques and an increasing number of patients being treated for an oncological condition. In the present case, the patient had incomplete paralysis and was treated with posterior decompression with internal fixation, which not only cured the MSCC but also gave a strong support for his spine.

RT is important in palliating the symptoms of patients with metastatic disease. The response to RT has been quantified and qualified with numerous criteria and instruments over the past decades (12). Studies have shown that 70–90% of patients achieve a beneficial response with analgesic-directed RT, with complete responses observed in up to 40% of patients (13,14). As the majority of spinal tumors are metastases, spinal RT is typically delivered using conventional two-dimensional or 3D conformal techniques, as demonstrated in the present case.

EGFR inhibitors comprise a novel, molecule-targeting treatment for lung cancer. These inhibitors have been reported to exert promising effects in females and nonsmokers, particularly those with adenocarcinoma (15–17). Treatment with an EGFR inhibitor may prolong survival following bone metastasis (8); however, interstitial pneumonia remains a serious side effect (18), and it has been reported EGFR inhibitors are less effective in patients without the EGFR gene (15). The indications for EGFR inhibitor therapy should therefore be considered carefully prior to expecting an improvement in survival. To the best of our knowledge, few reports have focused on EGFR mutations as effective prognosis indicators for patients with lung cancer adenocarcinoma metastasized to the spine (11,19,20). EGFR mutations should be verified for this patient group. In the present case, the combination treatment regimen of radiation and EGFR inhibitor therapy following surgical treatment was proved to be fully effective for the patient, as shown in the PET-CT images and by the good quality of life during follow-up.

In conclusion, the present study describes a case in which favorable results and a good recovery were achieved based on the selection of surgical treatment combined with radiation and EGFR inhibitor therapy, an option that is recommended for patients with lung cancer metastasized to the spine. We suggest that EGFR mutation be verified for patients with lung cancer metastasized to the spine. Furthermore, we encourage orthopedic oncologists to consider radiation and EGFR inhibitor therapy with surgery, based on the promising results observed in the present study.
